# Misconduct, Marginality and Editorial Practices in Management, Business and Economics Journals

**DOI:** 10.1371/journal.pone.0159492

**Published:** 2016-07-25

**Authors:** Solmaz Filiz Karabag, Christian Berggren

**Affiliations:** Department of Management and Engineering, Linköping University, Linköping, Sweden; Tilburg University, NETHERLANDS

## Abstract

**Objectives:**

The paper presents data on the two problems of misconduct and marginality in management, business and economics (MBE) journals and their practices to combat these problems.

**Design:**

Data was collected in three phases. First, all publicly retracted papers in MBE journals were identified through keywords searches in 7 major databases (n = 1329 journals). Second, a focused survey was distributed to editors involved in such retractions (n = 64; response rate = 28%). Finally, a survey was administered to all active journals in the seven databases to collect data on editors’ perceptions and practices related to the two problems (n = 937, response rate = 31.8%). Frequency analyses, cross tabulations, and qualitative analyses of open answers were used to examine the data.

**Results:**

184 retracted papers in MBE journals were identified in 2005–2015 (no retraction was found before 2005). From 2005–2007 to 2012–2015, the number of retractions increased by a factor ten with an all-time high in 2015. The survey to journals with reported retractions illustrates how already a few cases of suspected misconduct put a strain on the editorial workload. The survey to all active journals revealed that 42% of the respondents had started to use software to screen all submitted papers, and that a majority recognized the problem of marginality, as indicated by salami-style submissions. According to some editors, reviewers easily spot such submissions whereas others argued that authors may submit thinly sliced papers in parallel to several journals, which means that this practice is only discovered post-publication. The survey question on ways to support creative contributions stimulated a rich response of ideas regarding editorial vision, engaged boards and developmental approaches. The study uses data from three specialized fields, but its findings may be highly relevant to many journals in the social sciences.

## Introduction

In the natural sciences, research misconduct has been a serious issue for a long time, as documented in an analysis of 677 retracted papers from 1972 to 2006 [1]. The comprehensive retraction study by Grieneisen and Zhang [2] based on searches in 42 large databases from 1928 to 2010 detected retractions in 1,796 journals, with very few occurring before 1980, a modest growth in 1980–2000, and a tenfold increase in 2000–2010, also after the authors accounted for the general growth in publication volume and the effect of serial fraudsters.

Surveys directly targeting researchers suggest disturbing levels of misconduct and questionable research practices, especially when respondents are asked about their colleagues´ practices. According to a review of 21 comparable survey studies, 2% of the participating scientists admitted to have fabricated or falsified their research data at least once, and 14% answered they had ‘personal knowledge of a colleague who fabricated or falsified data’, with a much higher proportion of affirmative answers when queried about ‘questionable research practices’ [3].

Within the social sciences, in particular economics and business/management studies, research has focused on misconduct among students [4, 5], and there has been a tendency to avoid an open acknowledgement of misconduct problems among faculty. Thus only 30% of the editors in a survey to economics journals indicated that a public notice of plagiarism (a common form of misconduct) would be the most likely response when confronted with the question: ‘In a clear case of plagiarism, which of the following are appropriate responses?’ (page 490 [6]). Other studies give examples of outright denial when editors are facing suspicious papers: ‘Already burdened by professional obligations..... an editor´s typical mechanism of defense is denial……As there are no professional bodies to regulate and monitor editorial work, there are also no adverse consequences for editors to simply refuse to deal with research integrity issues.’ (page 553 [7]). In recent years, however, the growing evidence of research dishonesty and several revealed serial offenders have caused a change of mind. A survey of US business schools by Bedeian, Taylor and Miller (page 716 [8]) shows a widespread knowledge of colleagues engaged in plagiarism, ‘used another´s ideas without permission or giving due credit’. Honig and Bedi [9] reported that almost 1 of 4 papers at one of the major divisions of the Academy of Management in 2009 contained some degree of plagiarism. In a survey to European economists, large proportions of the respondents stated they had been involved in at least some questionable practices (page 5 [10]), ‘presented empirical results electively so that they confirm one´s argument” (32.2%) or “copied from your own previous work without citing’ (23.6%).

In addition to plagiarism and other dishonest behaviours, Bedeian, Taylor and Miller highlight another problem: repetitive publishing, using ‘the same data or results in two or more publications’ [8]. This issue has been discussed in management and business journals as a problem of increasing marginality, attributed to factors such as ‘incremental gap-spotting research’ (page 12 [11]); ‘an environment in which scholarship is increasingly mechanized and industrialized’ (page 367 [12]); an escalating focus on volume where ‘More is being produced but the big impact papers remain elusive…’ (page 6 [13]). In a survey to editors of Wiley journals (unfortunately only 7% of these were social science journals) 31% of respondents considered redundant publication, i.e. overlapping or ‘salami’ publication, as ‘a significant’ or ‘very serious problem’, scoring higher than plagiarism or undisclosed author interests (page 349 [14]).

Misconduct and marginality could be understood as interrelated problems: the acceptance of papers which should have been rejected, and the crowding out of potentially creative papers by incremental pieces trotting the beaten track [15]. Many MBE-researchers and editors emphasize that ‘scientific journals are keystones in the edifice of any serious discipline’ (page 337 [16]) and thus should represent the classical academic ethos, embodied in the CUDOS principles of commun(al)ism, universalism, disinterestedness and organized skepticism [17, 18]. However, there are very few studies of revealed misconduct in MBE journals, very limited data on how editors experience the problems of misconduct and marginality, or which practices they develop to deal with these problems.

Addressing those gaps, the paper investigates the trends of retractions in MBE-journals as well as editors´ perceptions of the marginality problem; practices in use in these journals to combat the problems of misconduct and marginality; and editors´ positive ideas regarding practices to support more creative contributions. The evolving discussion regarding problems in publishing has also identified a related group of issues labelled questionable research practices (QRP). A recent paper in Journal of Management lists several different QRP-elements: selectively reporting hypotheses, excluding data post-hoc, hypothesizing after results are known, selectively including control variables, and falsifying data [19]. Apart from ‘falsifying data’ which belongs to the category of outright misconduct, our study does not cover the questionable practices listed above, which, as several responses indicate are harder to deal with effectively from an editorial point of view.

## Method and Data

### Definitions of misconduct and marginality

Several definitions of academic misconduct exist. The US Office of Science and Technology Policy defines research misconduct as ‘fabrication, falsification, or plagiarism in proposing, performing, or reviewing research, or in reporting research results’, adding that this does not include ‘honest error or differences of opinion’ [20]. Some authors argue that fabrication (invention of data or cases), and falsification (willful distortion of data and results) are the most damaging forms of misconduct for the credibility of science, whereas plagiarism does not directly distort knowledge, but damage the scientific enterprise via its impact on careers [3]. This paper will use the terms research misconduct and academic dishonesty interchangeably as umbrella terms for intentional wrongdoing concerning all these forms, including self-plagiarism. In general, plagiarizing of other papers seems to be the most easily detectable form of misconduct. Self-plagiarism is harder to handle, although associations such as Academy of Management promulgate strict norms regarding this issue. In reality, however, each journal has to define acceptable levels of repetition without reference. Our study used public retractions as strong indicator of revealed misconduct, although a minor part of retractions are publically reported as “data errors” or mistakes. The problem of marginality is harder to delineate precisely. The editor survey II (see below) employed questions on “salami-publications” (slicing of output in least publishable units) to indicate this problem.

Few survey respondents seemed to have any difficulties to understand the meaning and their answers are used to indicate their perceptions of the problem. As mentioned above, ´redundant publication´ is another term used to indicate marginality. When required, the paper explicates the precise term used to indicate this problem.

### Study design and field of study

The study used a mixed method design, combining public data on retractions with two rounds of surveys to gather data on editors´ experiences of retractions, and their practices of dealing with the two problem of misconduct and marginality. The paper focuses on journals in three fields: management, business and economics (MBE). There are several reasons for including all three. First, journals generally belong to more than one subject category in the databases. Moreover, rankings of, for example, business schools include business, management as well as economics journals in their appraisals [21]. Other rankings follow similar paths, one example is Harzing [22] which includes management, marketing, finance, accounting, and economics.

Investigations of retractions in previous studies have focused on specific databases such as PubMed [23, 24, 25], specific publishers [14] or utilized multiple indexes and data bases [3]. In the present study, we used the databases from seven major publishers, which together comprise 1329 MBE-journals. The Web of Science database indexes 615 MBE journals and has been used for control purposes. See Table 1 for an overview of databases and journal numbers in the MBE category.

**Table 1:** List of databases in the retractions search.

Database	Number of MBE journals in the database
Emerald	347
JSTOR	278
Sage	71
SpringerLink	153
ScienceDirect	212
Taylor & Francis Online	96
Wiley Online Library	172
Total	1329*

*Of this number, 937 were identified as operating with active contact addresses at the time of Survey II (see “Data collection and analysis”).

### Data collection and analysis

The data in this study were gathered in three different phases. In the first phase we searched all publicly retracted papers in the MBE-journals included in the seven databases, without knowing the date of the first retraction. This date turned out to be 2005. Using the keywords Retraction, Plagiarism, Academic Dishonesty, Research Misconduct, Retraction Note, Retraction Notice, Retracted Paper, Statement of Retraction and Boolean strings including “retract*”, these searches uncovered 184 retractions in this period.

In the second phase we collected qualitative data on editors´ retraction experiences by means of a focused survey (Survey I) with open-ended questions developed by the researchers (see Table 2). In the paper development process, it was found that several of these questions were similar to the questions used in a study of retractions by Williams and Wager [26]. These similarities support the validity of the questions used in this study. The survey targeted 64 MBE-journals with at least one retraction which had been identified in the database searches. Before administering the survey, the questions were reviewed by one experienced editor who had been involved in several retractions at one leading journal. Eighteen editors responded, which implied a return rate of 28%, similar to the study by Enders and Hoover [6].

**Table 2:** Questions in Survey I.

*The records indicate your journal retracted a paper/papers in XXX*.
Have you suspected other submissions of plagiarism or manipulation recently?
How did you deal with this/these suspected case/s?
Who initiated the process?
What were the main steps in this case?
How much work was involved and how long did it take?
Which were the main difficulties?
How did you decide what to do?
How did the author/-s react?
What (if anything) did you change as a result of this process?

In the third phase we designed a survey to all the MBE journals investigated in the retraction search in order to capture perceptions, actions and ideas related to both the misconduct and the marginality problems. This second survey asked specific questions on a number of practices discussed in the broader science community: the application of screening software to detect plagiarism [14]; the use of replications [27, 28]; measures to specify co-author contribution [29, 30]; methods to assess and publicly reward reviewers [31]; as well as new techniques, including crowdsourcing, to reach out to untapped reviewer communities [32]. To gauge the prevalence of the marginality problem, the survey used questions on salami publishing as an indicator. Moreover, the survey sought to capture editors´ ideas on how to encourage more imaginative and generative contributions. Here the public discussion has not proposed any specific practices. Thus we asked in an open-ended way about suggestions and possible actions. All questions are available in S1 Table. The survey was pretested with 5 editors actively involved in misconduct and integrity cases. After eliminating journals which had ceased to publish, merged with other journals, did not disclose any useful editorial contact information or were clearly outside the MBE-field, we were left with 937 journals in the seven databases. For those with several chief editors, two or three respondents were listed. Defunct addresses and passive cases were omitted, resulting in a list containing 1197 editor names. After two reminders, 356 respondents had finished the survey, some of them from the same journal. Since reminding a respondent has been recognized as a forced answer [10], we kept the first answers received. Of responses within the same period, we kept answers with no missing data. The final count amounted to 298 journals and a response rate of 31.8%, similar to the response rates in other MBE-surveys. Table 3 summarizes descriptive statistics for participant journals, divided according to journal field and index status (ISI/non-ISI).

**Table 3:** Survey II descriptive statistics for journal population and respondents, %.

		Population after eliminating inactive/irrelevant journals %	Responding journals%
**Journal Field**			
	Economics	33	30
	Business	55	53
	Cross-Disciplinary	12	17
**Journal Indexing Status**			
	ISI	47	55
	Non-ISI	53	45
**Total number**		**937**	**298**

As can be seen in Table 3, cross-disciplinary journals (which include papers from management, business & economics) responded to a higher degree than they were represented in the overall population. Table 3 also shows that ISI-indexed journals responded to a somewhat higher degree than non-indexed journals: 55% of the respondents belong to the ISI-class compared to 47% in the population. Overall, however, the differences in response rate are small. Higher quality journals (indicated by their ISI-status) receive more submissions, which increase the burden on their review processes, but they also tend to engage more rigorous reviewers.

We present descriptive statistics for the answers to the close-ended questions (yes, no, I don’t know) to highlight the overall diffusion of editorial practices regardless of journal field and indexing. In the analysis, we also use cross tabulation and Pearson Chi square tests to capture potential associations between editorial practices and journal fields or indexing status (being an ISI or non-ISI journal). In all cases minimum observations ≥ 1. In one case (crowd sourcing) the observations in two cells were less than 5, therefore we used Likehood Ratio Chi square test instead of Pearson χ^2-^test. When the expected count was less than 10 in a 3X2 contingency table we used Likehood Ratio χ^2-^test instead of Pearson χ^2-^test to decide if the result was significant. If it was less than 20 in a 2X2 contingency table we also observed Fisher’s Exact Test to check significance level [33]. When a χ^2^ result was significant, we also checked the effect size: “For the reader to appreciate the magnitude or importance of a study’s findings, it is almost always necessary to include the measurement of effect size in the result section” (page 34 [34]). For 2X2 contingency tables we used Phi value to observe the effect size, for 3X2 contingency tables we used Cramer’s V for the same purpose [35].

The survey also contained four open-ended questions with free text answers. For several reasons, quantitative approaches to identify ´representative answers´ are of little value here: the highly varying response rate (from 48 to 163 comments); the huge variation in terms of substance and length (ranging from 0.5 to 19.5 lines in our printout), and the explorative intention of the questions. Formal analyses of qualitative data, e.g. content analysis (CA), may reduce variation into a set of numbers describing dominant themes and relative presence. This has been used in analyses of text documents, such as annual reports, although CA seems to be losing its appeal where it used to be the method of choice [36]. A strong tradition in qualitative research emphasizes the importance of avoiding reductionism, and listen ‘to the subjective experience and stories of the people being studied’ (page 26 [37]). This tradition advocates close reading, interpretation and quoting to convey the richness of the collected data, and suggests methods that combine transparency with ‘the necessary degree of intuition… that make the analysis creative and fruitful’ (page 12 [38]).

Building on this tradition we analyzed the free comments in the following way: After a basic check of the number of eligible comments and their most frequent themes, the authors independently reread and classified all comments, marked which of them to use for illustrative quotes, compared their choices and agreed upon a final selection which would illustrate important experiences, analytical observations and diversity of ideas; the last aspect was particularly important for the survey question on ideas to encourage creative contributions.

The presentation below includes a rich commentary to all open-ended questions. S2 Table provides an overview of the number of open comments to these questions. As noted above some comments were very short and of little informational value; others included substantial experiences and analyses. Comments exceeding three lines in our printout (on average 20 words) qualify as ´rich´ and are the main source of the selected quotes, since length turned out to be good proxy for richness of reflection. If the original comments were very long we selected the most relevant part (excluded parts are indicated by dots at appropriate places in the quote; the full comments can be found in data files in the supporting information). In exceptional cases, when all respondents offered very brief answers, we also quote from such short comments. That is the case for answers regarding crowdsourcing techniques. We also quote from shorter comments when editors responded by a request for advice, as some did regarding practices to support more creative contributions.

An overview of the three data collection phases, the retraction study, the survey to journals involved in retractions, and the second survey to all MBE-journals, is provided in Table 4.

**Table 4:** Overview of data collection phases and methods.

Phases	Aim	Data source and numbers of units searched	Method	Analysis & Results
**1**	To find all publicly retracted papers in management, business and economics journals	Seven major databases, covering MBE-journals (see Table 1).	Database search using Boolean strings Retract and the keywords Retraction, Retracted, Plagiarism, Academic Dishonesty, Research Misconduct, Retraction Notice, Retracted Paper, Statement of Retraction Timing: Last search April 2016.	Data coded in an SPSS list. Frequency analysis of reasons for retractions, etc. Total number of retracted papers: 184 between 2005 and 2015.
2	To explore the experiences of editors involved in such retraction processes.	Survey I sent to 64 journals with at least one retracted article (early 2014).	Focused survey with mainly open-ended questions. See Table 2. Editors contacted via e-mail Timing: Mail to the editors, two reminders, Jan.–Feb. 2014.	Editors at 18 journals responded. Response rate: 28% Qualitative analysis of free text answers—close reading, interpretation
3	To examine the diffusion of editorial practices related to misconduct, perceptions of marginality, and ideas to encourage creative publications.	Survey II: Target population 1329 journals. After removing inactive/irrelevant entries, survey was sent to 1193 editors, representing 937 journals	Survey questions based on literature and pretested with 5 editors involved in misconduct and integrity issues. They include background questions, several close-ended questions on editorial practices, 2 comments areas and 2 open-ended questions. Survey sent via https://sunet.artologik.net/. Timing: The survey was online between 17th of July and 10th of October, 2014.	The survey was started 399 times and was finished 356 times. Eliminating duplicate answers and answers with too many missing data resulted in 298 useful and complete answers. Response rate: 298/937 = 31,8% Frequency analysis; cross tabulation and Chi square test, Qualitative analysis of free text answers—close reading, interpretation, classification, selection.

### Limitation of the Research

The main limitations in the study are as follows. The first limitation is related to the method to identify retraction trends. In our database search for retractions we used a number of keywords related to “Retract”. As Grieneisen and Zhang [2] note, journals often use ´softer’ terms for retracted papers. Such terms are difficult to identify, and our search probably underestimates the real extent of retractions. Another limitation is related to the selected databases. They have a comprehensive coverage of the relevant fields, but it is difficult to know the extent of non-coverage. Using these databases limits the search to journals published in English, moreover these databases mostly cover journals with authors from developed countries. To overcome some of these weaknesses Business Source Premier and ISI were checked for control purposes and to find missing retractions.

The third limitation is related to the semi-confidential nature of the retraction process targeted in Survey I. Several editors mailed us to inform they had discussed the survey with other editors and decided not to participate because of the sensitivity of the issue, and the risk of revealing confidential information about individuals. These concerns limit the representability of the survey in similar ways as reported by previous studies [6, 26]. However, it can be argued that when an editor answered our survey s/he had made a considered decision to share his/her experiences and this contributes to the overall reliability of the information. Another limitation of this study is the number of respondents (18). However other published studies have made credible arguments based on smaller number of respondents [26]. It should also be noted that we systematically targeted the editors who actually managed the retractions. Each retraction announcement was analyzed and the retracting editor identified. On the basis of these identifications the survey was sent to respondents irrespective of their current role at the targeted journal. Some retired editors chose not to participate, but the data we did receive could be trusted to exhibit a high internal reliability. The reliability is further strengthened by the similarity of our findings to the results from other studies of editors involved in retraction processes [26].

Both our surveys collect data with self-reporting tools, e-mails (Survey I), an online questionnaire (Survey II). In order to increase participation survey II was done anonymously. Previous studies show that as time goes by negative memories fade faster than positive ones and individual recalls become less precise [39]. There is also a tendency that social desirability influences the response to some type of questions [40]. On the other hand, this is the only way to collect the data in many cases [41]. Moreover, management studies document a strong overall correlation between subjective and objective measurements [42]. When possible, answers are compared with other sources. In contrast to personal interviews, surveys do not allow the researchers to ask respondents how they interpret various questions. In our study, the free text comments had the additional value of providing insights into these issues. However, these qualitative data also implied a challenge for the analysis. As explained above, we used close reading and extensive quoting to capture the richness of these data. Whenever possible, quotes were selected from lengthy comments, where the respondents explained and elaborated on their experiences. Interpretive methods make richer use of the data than reductionist ones, but are criticized for the difficulties they pose in replication. Selections may imply bias, and other researchers might select quotes in a somewhat different way, but if they followed the same ground rules of giving voice to variety and contrasting views, the basic pattern would be similar. Informed selection and categorization based on clear cut-off criteria are necessary in all types of research [43]. In this case, the PLOS requirements to make supplementary materials available, including all respondent comments, reduce the risks that bias will go undetected by readers.

## Results

### Retraction patterns

**Fig 1:**
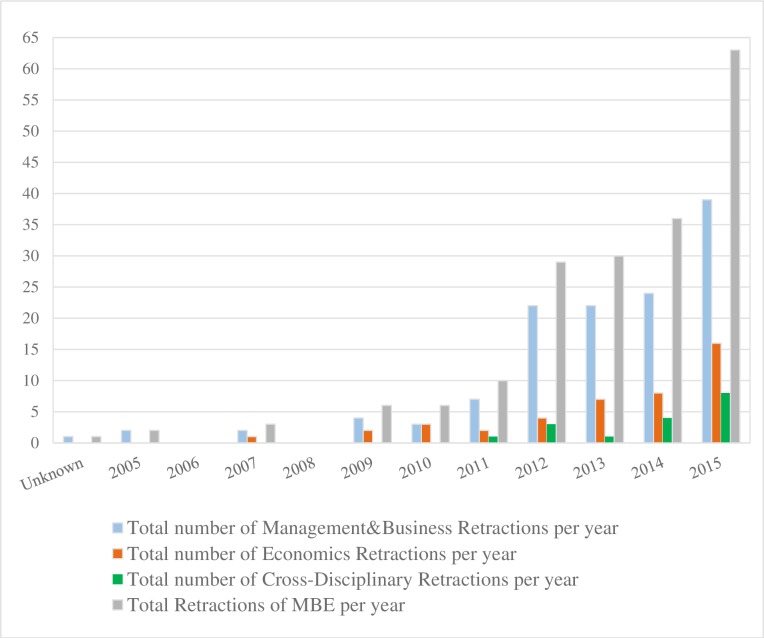
Total Numbers of Retractions in Management/Business and Economics from first documented retraction until the end of 2015. (One retracted paper was published in 2001, but retraction year is unknown.) Sources: Authors calculation based on keyword searches in seven databases.

The database search for retractions identified 184 retracted papers in the 2005–2015 period: 125 in management/business journals, 43 in economics journals and 16 in cross-disciplinary journals (see Fig 1). Of the stated retraction reasons, 38% referred to plagiarism/self-plagiarism, another 27% to fabrication and falsification, 9% to data and statistical errors, and 7% referred to fake reviewer. An 8% of total retractions did not state any reason. For more information about the reasons of retraction see Table 5.

**Table 5:** Retraction reasons of the papers in MBE from first documented retraction until the end of 2015*.

Type of retractions	Frequency	Percent (%)
Plagiarism	41	23
Self-Plagiarism	27	15
Fabrication	48	26
Falsification	2	1
Problematic data	10	5
Statistic error	7	4
Duplication	12	7
Author administrative error	2	1
Others (Publisher + Editorial)	3	2
Ethical problems	2	1
Fake reviewer	14	7
Unknown	16	8
Total	184	100.0

*Sources: Authors´ calculation based on the retraction notes published in the databases

**Fig 2:**
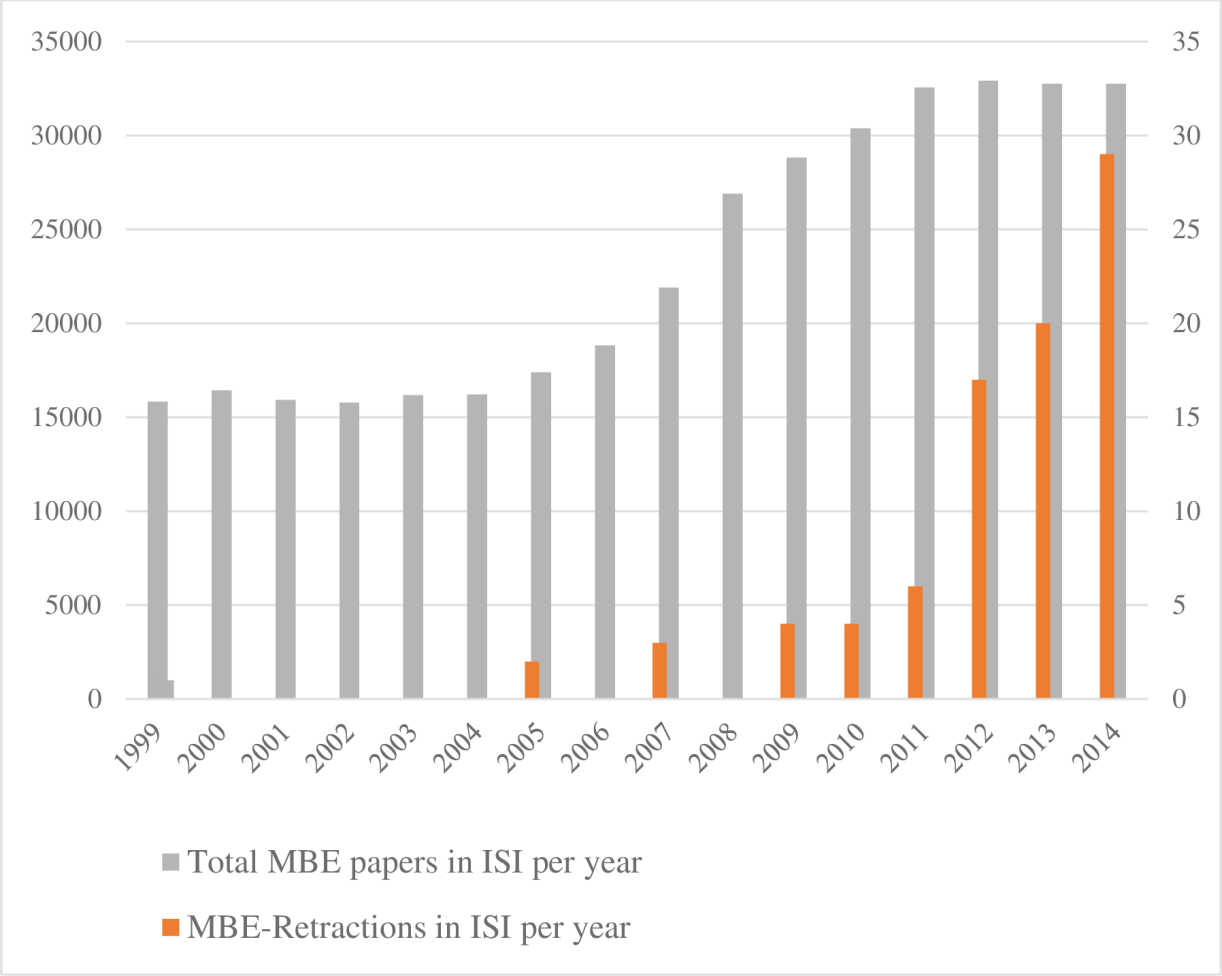
A comparison of total Retraction in Management, Business and Economics and total number of retractions in ISI journals. Total ISI (SSCI) publications in Management, Business and Economics per year (left axis). Total number of retractions in ISI journals (right axis) between 1999 and 2014.^1 1^A paper with no retraction year is not counted, although that was an ISI journal paper. Sources for retraction data: Authors´ calculation based on keyword searches in seven databases.

The predominance of plagiarism seems to be an indication of publication pressure, which has been ‘found to be positively related to the admission of being involved in several unaccepted research practices’ (page 1747 [10]). As Fig 1 illustrates, the trend of retractions is increasing with a possible acceleration in the last few years. The trend remains the same when we account for the growing volume of published papers. During the studied period there has been a tenfold increase in retractions in ISI-journals versus a doubling of the total number of ISI papers published, see Fig 2.

Similar to the observation by Grieneisen and Zhang [2], our data show that the impact of serial offenders does not invalidate the observation of a general increase in retractions. The data for 2015 indicate that this year is a new “all-time high” with 63 retractions, compared to 36 retractions for the full year 2014, but this record figure may be excessively influenced by two serial fraudsters, one forced to retract 15 (!) papers in a single economics journal, the other being retracted in an array of accounting journals. Irrespective of the interpretation of the retraction trend–as a sign of increasing misconduct or as a sign of increasing vigilance—the growth in retractions and the public discussion have spurred the attention to misconduct problems among editors in the previously dormant MBE-fields. This is reflected in the diffusion of new practices, as described below. Compared to management and business, there are relatively few retractions in economics, although the trend is increasing as can be seen Fig 1. Answers in Survey II indicate several concerns among economics journals; one editor commenting in the following way:

“The problem I see in the field of economics is that we have gotten used to checking the quality of the prose and references and mathematical derivations, but we never checked data and empirical methods. I do not expect that a large majority is knowingly ´dishonest´ but I expect that there is a larger share of authors which is unknowingly quite lax when it comes to data and methods. My feeling is that data and methods should be subject to the same scrutiny as a mathematical proof…”

### Editor experiences of retractions (Survey I)

Survey I targeted MBE-journals with identified retractions as of early 2014. Although the study did not seek to capture the trend of suspicious submissions exposed before publication, half of the responding editors reported they had detected or suspected other cases of misconduct before publication, for example:

“A critical case now, when there is a 30% overlap through the paper. The author has also applied for a position at a University in /country X/ which has evolved into a legal battle.” (Management journal).

Answering the questions regarding retraction experiences—“How much work was involved?” and “Which were the main difficulties”—some reported curtly:

“A lot of work, pls don´t ask me to quantify.” (Economics journal).

“A lot of work. It took about four months. I probably spent 50 hours on it.” (Business journal).

Other responses contained detailed descriptions:

“In this case, the author did not contest legally, but still it was horrendously burdensome. A research assistant had to go through 15 papers and then two reviewers looked independently at everything. Thereafter a mail with detailed questions to the author re lack of self-referencing and methodological inconsistencies, and the author responded with a 12 pages (!) letter. At the end we retracted two papers and informed his employer.” (Cross-Disciplinary journal).

“It involved reviewing other articles published by the author (those cited in the papers published in X), comparing the articles with regard to data sources and construct measures. We also requested the original data files from the author because of statistical inconsistencies. There were many e-mails, several phone conversations over a period of about 2 months. We also examined other articles by the author that had been retracted from other journals. We also corresponded with the author via e-mail.” (Management journal).

“The difficulty starts early on. With authors publishing in so many journals these days, an Editor cannot expect volunteer reviewers to spot every instance of plagiarism, fraud or copyright violation. Then there is the frustration of having to take time away from processing the papers sent by ethical authors in order to deal with the problem author. Then waiting for the Publisher’s retraction committee to take its own sweet time in responding. Finally, the embarrassment of having to retract a paper and apologize to readers.” (Cross-Disciplinary journal).

These testimonies indicate that relatively few cases of suspected misconduct might stress an already strained system and erode the editors´ possibilities to spend time on the curating approaches advocated below. Although it is wise to be careful when extrapolating from small samples, our approach is supported by other studies. Arguing that their selection of five editors from different fields “were broadly representative of the entire population”, Williams and Wager (page 4 [26]) emphasize the burden which retractions create on the system: “every retraction is a different case, and so it is not possible to follow one particular system…. the unique nature of some of the cases made it extremely difficult to develop even a framework procedure from which to work” (page 7 [26]). In addition to the lack of a standard procedure, the authors also note the risks of unforeseen complications regarding co-authors, copyrights, etc. which also add to the burden on editors.

### Editorial practices related to misconduct and marginality (Survey II)

As a result of the increased attention to misconduct problems, several MBE-journals have announced policies regarding plagiarism and other forms of misconduct. Survey II therefore started with a section containing specific questions regarding practices to detect and combat dishonesty. Table 6 provides an overview of the answers from the 298 participating journals. Chi-square tests were used to uncover possible associations between editorial practices and journal field, or indexing status.

**Table 6:** Diffusion of practices related to misconduct management (%).

Questions	No (%)	Yes (%)	I don’t know (%)
Do you regularly use software to check submissions for originality?	54.0	42.3	3.7
Do you ask authors to provide data files and calculations?	67.5	30.5	2.0
Do you request corresponding authors to provide information on the specific role of each author?	92.0	5.7	2.3
Does your journal have any policy regarding maximum number of papers/year authored or co-authored by any member of the editorial or advisory boards?	75.5	17.5	7.0
Do you experience any tendency of “salami publishing” (the slicing of output into least publishable units) in submitted papers?	41.6	51.7	6.7

In addition to answering the specific close-ended questions, 75 respondents offered free text comments; of these 15 related to the use of screening software and 13 to the salami issue. Of the total number, 21 contained longer statements, defined as ´rich comments´, including experiences of the problems, mini-cases, reflections and /or suggestions.

The survey shows that using screening software, such as “Ithenticate” or “CrossCheck”,to check submissions for originality is the most frequently used practice to combat plagiarism: 42.3% of the journals state they use such programs to inspect submissions before sending them to reviewers (see Table 6). According to the chi-square tests there were no statistically significant associations between journal field and the use of software to check submissions, χ^2^(2, N = 287) = 4.14, p = .13; or between a journal´s indexing status and the use of software, χ^2^(1, N = 287) = 0.24, p = .63. See S5 Table for the cross tabulations.

In the free text area several respondents report they are “in the process of installing software” or “the option is available”. However, respondents point out that the screening results must be evaluated intelligently:

“….Plagiarism software should not make decisions, editors should.” (Economics journal).

“We use iThenticate to scan all submissions deemed reviewable. Before final acceptance, all papers will be scanned again to make sure no more than 10% of similarity to published work.” (Management journal).

The widespread use of screening in the pre-publication process should reduce the number of manipulated papers appearing in print. Some journals, however, report difficulties to apply these tools to detect questionable practices:

“Plagiarism is a common problem, but it can be detected with appropriate software. However we see more and more papers coming from some countries which just repeat in their own words what has already been published in mainstream journals a few years back.” (Management journal).

Comments to the open survey question (“Please share ideas or practices which may help to reduce the risk of dishonest papers being published “) also addressed the limitations of screening software:

“I received a review ms from Country X in which every single line had been published, usually in an Abstract. It was a great review and surprisingly readable, I could not though persuade the author that this was plagiarism, as every paper had been cited. Honest, dishonest, cultural difference? I have no idea.” (Cross-Disciplinary journal).

“I have had a case as an editor in another journal where some authors from Country Y re-worded a paper excellently but when I searched online, I found very relevant people. They complained that the authors had completely used their work by using their equations but re-wording and paraphrasing terms (so software was unable to verify plagiarism).” (Economics journal).

Revealing manipulations in a submission before publication, instead of retracting a published paper, intuitively seems to be the best way to deal with dishonesty. However, the lack of public information and generalized sanctions may work in the opposite direction, as one respondent comments:

“Sometimes, after having exposed plagiarism and rejected a paper we find it published, uncorrected, in another supposedly respectable journal. We suspect their editors/reviewers are overworked and try to get things done too quickly.” (Management journal).

Another respondent observes:

“The large variety and diversity of journals and conferences that are available now result in ´everything being published´ irrespective of originality and quality….” (Management journal).

The second question in Table 6 asked respondents if they require authors to supply data files and calculations. Less than a third of all journals answered positively (See Table 6). The chi-square test shows a statistically significant association between journal field and this question: χ^2^(2, N = 292) = 30.89, p < .001; a Cramer's V value of 0.33 (p< .001) indicates a moderate level of association [33]. While 46 of 84 economics journals ask authors to provide data files and calculations, only 31 of 158 business & management journals and 14 of 50 cross-disciplinary journals do the same. However, no statistically significant association could be found between requiring authors to supply data files and calculations and journal indexing status, χ^2^(1, N = 292) = 0.77, p = .38. See S6 Table for the cross tabulations. The analysis indicates that economics journals require submission of data and calculations more often than management/business and cross-disciplinary journals, which may be related to the use of more public data sources in economics papers. However, an editor points to increasing problems regarding this practice:

“I think publicizing the data is the way to go. But these days there are so many proprietary and confidential data; it is difficult to consistently carry out such policy.” (Economics journal).

Another editor emphasizes the subtle problem of ´data massaging´:

*“*I believe that most are not dishonest, but do data massaging because the data does not say EXACTLY what they want the data to say. … Given the fine tooth combs we use to evaluate the statistics and the lack of tolerance for any type of odd result, I believe the 'system' may in fact encourage data massaging. … We need to recognize that in social science, odd results are possible; nothing comes out perfectly; and such results should not be a reason for rejection.” (Management journal).

The third question in Table 6 concerns the role of a paper´s co-authors. The problem of co-authors, who bask in the glory of publications but evade responsibility when a manipulated paper is exposed, is discussed in several reports on revealed offenders, such as the Dutch social psychologist, Diederik Stapel [44]. In our survey only 5.7% of the respondents indicated that their journal requires co-authors to state their specific contributions (see Table 6). A χ^2^-test was performed to examine the relation between answers to this survey question and journal field, but did not find any statistically significant association, χ^2^(2, N = 291) = 1.91, p = .38, nor any significant relation between this question and journal indexing status, χ^2^(1, N = 291) = 0.42, p = .62. See S7 Table for the cross tabulations.

In the commentary section several editors qualified their answers: this is only applied “sometimes” or “if the journal has any questions about the role of an author”. One editor, however, took a stronger stand:

“I like the idea of asking corresponding authors to provide information about the specific role of each author. I am currently dealing with a case of possible fraud and some of the co-authors are claiming ignorance which does suggest that it is a good idea to know a- priori who knows what.” (Management journal).

The fourth question in Table 6 concerns limits for submissions from editors to reduce the risks of insiders crowding out contributions from less established academics. Only 17.5% of the respondents reported any kind of limit for the editors (see Table 6). The results of the chi-square tests indicated no statistically significant associations between journal field or indexing status and policy regarding limits for submissions from an editor, χ^2^(2, N = 277) = 1.99, p = .37; χ^2^(1, N = 277) = 0.23, p = .64. See S8 Table for the cross tabulations. Several free text comments pointed out that special journal issues may pose particular problems:

“Special issues are often full with dishonest practices. Recently, a guest editor of a special issue of 3* journal inserted her name on the papers and by which she was offered a professorial post by a dean who is also practicing the same dishonesty. I assume both will repeat the same fraud in near future.” (Business journal).

“One of the challenges is to avoid special issues in which a group of authors referee each other so that a group of papers ends up being accepted ´as a group´. This unfortunately happens occasionally, and I can point to specific instances where it has happened.” (Management journal).

In addition to the questions on specific practices, an open survey question asked respondents to share “ideas or practices to reduce the risk of dishonest papers being published”. This elicited 163 comments, 62 classified as rich. Of all comments, 22 concerned software screening and its limitations (two are cited above) and 30 addressed reviewer issues, indicating that the review system still commands a central place:

“I rely on strong reviewers to screen for dishonest papers. This process has resulted in screening out over a dozen papers in the past decade.” (Management journal).

“We depend on our reviewers to let us know if they have seen manuscripts in print in other places. That is clearly not the most efficient method, but it has worked to date.” (Management journal).

Regarding other ideas to reduce the risks of publishing dishonest papers, a few comments mentioned the role of the editorial board; one referred to their journal being a member of COPE with access to its Guidelines and Code of Conduct; another mentioned the journal´s Code of Ethics with sections for authors, editors and reviewers to sign up. Several comments involved sanction policies, implemented or wished for:

“…In these cases/of plagiarism/, the papers are rejected and authors are informed that further submissions from them will not be considered for publication by the journal.” (Business journal).

“Make the costs higher. Too often just the paper is rejected. Journals should copy the rejection letter… to the submitting author´s dean or department chair.” (Economics journal).

In addition to misconduct problems editors also perceive a problem of marginality in submitted papers. Of all respondents 51.7% stated they had encountered instances of “salami-publishing” (see Table 6). Chi-square tests of the association between perceptions of “salami publishing” and journal field or indexing status did not indicate any statistically significant results, χ^2^(2, N = 278) = 2.91, p = .23; χ^2^(1, N = 278) = 0.58, p = .45. See S9 Table for the cross tabulations.

Salami issues were discussed in 13 of the 75 open comments to the five survey questions in Table 6. One editor related the problem to the performance demands in many universities:

“Every university is pushing publishing so hard that this results in significantly lower quality research in total. … From an editorial perspective, the vast majority of the work is derivative, or makes observations that amount to ´this tiny square of the sky is blue #87362a unlike the sky over there which is blue #87362b´." (Economics journal).

Another comment pointed to a similar analysis:

“The use of ´metrics´ in promotion and tenure decisions is pervasive, in all but the top universities. In xx, professors' teaching loads are (or at least used to be) reduced according to the number of papers published. Salami publishing was the result. Especially in economics, faculty members respond to incentives.” (Cross-Disciplinary journal).

Several editors reported they use screening software also to identify salami or redundant publications, whereas others found reviewers to be good at spotting salami submissions: “Salami, those are easy rejections, we note that the innovation is not sufficient” (Economics journal). The opinion is split, however. One economics editor commented that the question is difficult to answer: “…The issue is in a murky state” whereas another found the issue almost insurmountable:

“The problem of 'salami publishing' is difficult. Invariable when one idea is split into three, the other two parts are submitted to journals outside the group of journals managed by my publisher. End result is that I know they are doing it, but can’t catch them until all is published and it’s too late.” (Management journal).

The extent of and solutions to this issue seem to warrant future investigations.

### Editorial practices related to the review systems (Survey II)

As seen above, journals still rely on their reviewers (with some support from screening software) regarding both the misconduct and marginality issues. A survey section on editorial practices included four questions on reviewer quality and recruitment, as well as questions on replications and invitations to debates. See Table 7. These survey questions were followed by a free text option which elicited 48 comments, whereof 17 classified as rich. Most of them discussed problems of finding and maintaining good reviewers (See S2 Table for the questions).

**Table 7:** Journal practices related to reviewers and the review process.

Questions	No (%)	Yes (%)	I don’t know (%)
Do you have any public rewards for good reviewers?	60.7	37.9	1.3
Do you have any policy to add good reviewers to the advisory board after specific years?	44.6	49.3	6.0
Have you tried to implement any crowd-sourcing techniques to engage more reviewers?	91.9	5.7	2.3
Has your journal published any replication studies in the last two years?	72.5	10.4	17.1
Do you use any review quality instrument to engage authors in evaluating the reviewers?	77.9	19.8	2.3
Has your journal recently organized debates on a specific theme or finding?	49.7	48.0	2.3
^n^ = 298 journals			

As Table 7 shows, 37.9% of the responding journals state they have public rewards for good reviewers, including ‘best reviewer awards in progress’. No comments give any details, except one which briefly refers to Emerald´s recognition system. The chi-square test uncovered a statistically significant relation between journal field and rewards for good reviewers, χ^2^(2, N = 294) = 25.55, p < .001. The effect size for the relation was moderate, Cramer’s V = 0.29 (p<0.001). While 80 out of 158 business & management journals (50%) use rewards for good reviewers, 16 out of 85 economics journals and 17 of 51 cross-disciplinary journals use similar practices. On the other hand, no statistically significant association between rewards for good reviewers and journal indexing status was observed, χ^2^(1, N = 294) = 0.07, p = .79. See S10 Table for the cross tabulations.

Almost 20% of the respondents state they use review quality instruments (RQI) (see Table 7). No statistically significant association between this practice and journal field or indexing status was detected, χ^2^(2, N = 291) = 2.61, p = .27; χ^2^(1, N = 291) = 0.56, p = .45. See S11 Table for the cross tabulations. In the absence of any free text comments on this issue, it is hard to interpret what the positive answers from the 20%-minority mean and what sort of RQIs these journals implement.

Almost 50% of the journals report they have a policy to acknowledge good reviewers, but no comment mentions any published journal policy to promote reviewers to official journal roles (see Table 7). The chi-square test indicates a statistically significant relation between journal field and this practice, χ^2^(2, N = 280) = 21.23, p < .001, with a moderate effect size, Cramer’s V = 0.28 (p < .001). While almost two thirds (98 out of 151) business & management journals are likely to add good reviewers to the advisory board, 28 out of 81 economics journals and 21 out of 48 cross-disciplinary journals have this practices. On the other hand, no statistically significant association between this practice and journal indexing status was observed, χ^2^(1, N = 280) = 0.86, p = .35. See S12 Table for the cross tabulations. Several free text comments indicate that their ´policy´ is an informal practice of an ad hoc-character, as stated in a typical comment:

“No fixed policy of adding good reviewers to the advisory board, but tendency to do so…” (Business journal).

At the same time, several respondents report acute difficulties to recruit good reviewers:

“It is incredibly difficult to find and recruit good reviewers. … Further, most potential reviewers see the job as too much work and little reward.” (Economics journal).

“My most dismal record …contacting 13 potentials to obtain a single review.” (Cross- Disciplinary journal).

The literature suggests several new techniques, e.g. crowdsourcing reviews by publicly posting abstracts and inviting voluntaries from the relevant community, who are then screened and selected by the editor [32]. Only 5.7% of the journals report use of such techniques—with mixed results (See Table 7).

Three open comments addressed this issue, two of them distinctively negative:

“The crowd-sourcing does not work efficiently. I have tried but it led to wrong matches.” (Economics journal).

“The crowdsourcing idea is sexy, but it is so hard to get capable, reliable reviewers that it probably would become a ‘market for lemons’." (Management journal).

One editor, however, was highly positive:

“Just put out a call for per reviewers via social media and had an amazing return!” (Management journal).

Unsurprisingly, the chi-square tests did not display any statistically significant associations between crowd-sourcing techniques and journal field or journal indexing status: χ^2^ (2, N = 291) = 4.43, p = .11; χ^2^(1, N = 291) = 0.86, p = .46. See S13 Table for the cross tabulations.

Whereas journals in other fields, for example Brain and Behavioral Sciences, have developed ambitious systems for open commentary and author responses, MBE- journals seem hesitant to test new approaches which would involve more editorial work but also build stronger community responsibilities. Despite difficulties in recruiting and maintaining reviewers, these journals stick to conventional procedures, with the possible diffusion of reviewer awards as the most promising new practice.

One question in Table 7 specifically targeted replications. For decades there has been a debate regarding the need for more replications in business and economics research [45, 27]. In our survey, 10.4% of the respondents reported that a replication study had been published in their journal in the last two years (see Table 7). The chi-square test result showed no statistically significant relation between journal field and publishing of replications, or between indexing status and publishing of replication: χ^2^ (2, N = 247) = 0.83, p = .66, χ^2^ (1, N = 247) = 2.18, p = .18. See S14 Table for the cross tabulations. The positive answer from 10.4% of all respondents may seem low in relation to the many calls for replication studies, but high compared to other evidence. A study of the websites of 333 economics journals found that only 3 percent of them explicitly stated they publish such studies (page 174 [46]). The discrepancy between this figure and our survey may be explained by social desirability (editors know that it is desirable to publish such studies and maybe plan to do so), or by different interpretation of the meaning of ´replication´. Only two short free text comments mentioned replications, one of them referring to a replication study being ´in press´. Despite the acknowledged importance of replications for building robust theories and expose manipulations, editors´ interest in increasing the publishing opportunities for replications remains unimpressive.

Parallel to this question we also asked if journals organize debates on a specific theme or finding; 48% of the journals stated they do so (see Table 7). A chi-square test result indicated no statistically significant association between the answers to this question and journal field or indexing status: χ2 (2, N = 291) = 3.98, p = .14, χ2 (1, N = 291) = 0.03, p = .88. See S15 Table for the cross tabulations.

### Suggestions to increase publishing of more creative papers

If good practices to detect and reject manipulated or marginal papers are one side of the coin, practices to encourage and support more creative and impactful contributions are the other side. This aspect, however, is seldom discussed in the literature, and few approaches have been suggested. The survey´s open question “Please share ideas or practices which may help to encourage creative and thoughtful contributions” received suggestions from 98 respondents, almost half of them rich. Seven of the 98 comments referred to a standard practice (special issues). All other ideas had fewer proponents, an indication of the range of suggestions. If diversity is seen as a breeding ground for creativity [47, 48], this lack of ´representative statements´ is encouraging. Future studies may investigate the actual implementation of suggested practices.

In Table 8, the suggestions are consolidated into 14 themes, each with an illustrative quote, and the themes are further grouped into four major classes. Instead of suggesting ideas, several respondents reached out for help:

“Do not have a clear idea, please let me know your results.” (Economics journal); “I would be grateful to receive ideas from your results here.” (Cross-Disciplinary journal).

**Table 8:** How to support creative papers–fourteen themes and illustrative quotes.

Label	Themes	Illustrative quotes
Editorial vision and engaged boards		
	Open up, take risks	“Editors need to open up to new researchers and new concepts. . . far too many editors are just monitors/curators of historical artefacts and thought and thus much of my discipline is becoming moribund and lacking experimentation. . . . be thankful that science or medicine do not suffer the same narrow view!” (Business journal).
	Visionary editors	“Editors need to have clear ideas about what they want to see and should not be afraid to overrule everyone…the problems that you see are a result of sloppy editorial practice, where editors act only as an anonymized postal service between referees and authors.” (Cross-Disciplinary journal).
	Strong editorials	**“**One way is to develop thought provoking editorials in a hope to stimulate potential authors' creative research ideas. But I'm not sure how many people actually read editorial pieces.” (Management journal).
	Engaged editorial boards	“Editorial board members should not be a kitchen list of people. Rather they should not only have relevant experience but also should be interested in actually giving time to the journal. Very high profile people often do not have time to get reviews conducted or even reply to emails in time.” (Economics journal).
	Change editorial teams	“I think that changing editorial teams in a regular basis is good, and the most important thing is to look for representativeness of different perspectives inside the field in this editorial teams.” (Business journal).
Curate papers and connect authors		
	Curate and develop the manuscripts	“If research idea is of interest then editors and reviewers are expected to work with author(s) to feature the idea….Goal is to mentor and guide authors to maximize potential impact of paper.” (Management journal).
	Connect authors	“Encouraging dialogues between authors. Keeping the reviewers interested and also encouraging them to write papers….” (Business journal).
	Constructive screening	“Initial screening of each submission by editors (and maybe immediate revise and resubmit) to prevent creative but badly written papers from being rejected immediately.” (Management journal).
Open up for discussion		
	Publish criticism instead of rejection	“In case of controversial papers (unexpected findings, bold theoretical claims), invite hard-to-convince reviewers to publish a comment on the paper rather than reject the paper because one or two reviewers remain stubborn. This will spur debate rather than kill a potentially great germ.” (Business journal).
	Invite comments	“I like the Brain & Behavioral Sciences model—an invited/ competitive contribution followed by 5–20 commentaries/ critiques by a diverse set of commenters.” (Management journal).
	Involve industry specialists	“We …particular welcome comments from industry specialists. There is a strong industry circulation which helps keep the research relevant.” (Management journal).
Go beyond the mainstream		
	Mix disciplines	“Allow a brief state-of-the-art, be open for rough but non-mainstream research, and ask for papers with disciplinary mixed authors/theories/methods.” (Management journal).
	Avoid perfection	“…The only way to encourage creative and thoughtful contributions is to publish them even though they are not perfect.” (Cross-Disciplinary journal).
	Limit individual authors	“We also encourage new work from new researchers and try and avoid the 'same old names' who are just looking to increase their publications list for REF or Tenure purposes. . . I would much prefer to have a new researcher who challenges the norms than an old one who is playing a game!” (Business journal).
Source: Authors own data collection from Survey 2 question: *“Please share ideas or practices which may help to encourage creative and thoughtful contributions”*		

## Discussion: Interpretations and Implications

This paper investigates two problems in current publishing within management, business and economics. Starting with the problem of misconduct (fabrication, falsification and plagiarism) the study documents an increasing trend of public retractions in MBE-journals, from 0 before 2005 to around 30 per year in 2012–2014, with an all-time high of 63 retractions in 2015. (This year, however, is probably exceptional due to the exposure of two serial offenders, one in accounting and one in economics.) Plagiarism is stated as the most common reason. Noting a considerable number of hidden retractions reported as ‘errata’ or ‘corrected and republished’, Grieneisen and Zhang (page 16 [2]) concluded: ‘thousands of these ´implicit´ retractions exist in addition to the 4,449 ´explicit´ retractions in the dataset used here’. Similar problems are likely to exist in our retraction data. Moreover, several authors highlight the disincentives for the victims of misconduct, the plagiarized authors, to report such incidents. This further reduces the publically reported retractions [49, 50, 51]. Everything else being equal, the dissemination of software to catch suspicious cases of plagiarism in the pre-review phase should reduce the incidence of publishing this type of manipulated papers, and by implication, the frequency of post-publication retractions.

How then could the consistent increase be interpreted? Some observers of retractions in the sciences suggest that the trend may be explained by a growing number of journals issuing retractions, arguing that ´the editorial community´ as a whole has become more vigilant [52]. However, one could argue that if misconduct is on the increase, the number of journals involved would naturally grow. Ideally, our study could have tested the increasing vigilance-hypothesis by comparing retracting journals with journals without documented retractions. Unfortunately the number of respondents in the retractions group was too low to allow any statistical analysis. Irrespective of interpretation, the revealed extent of misconduct remains small compared to the total publication volume. Its impact on trust in science is potentially considerable, however [1] and the introduction of countermeasures, such as plagiarism-detecting software, increases the editorial workload to the detriment of time for developmental activities.

The second concern of this paper, the flow of marginal submissions, is indicated by a widespread editorial perception of salami submissions. The absence of longitudinal surveys makes it difficult to track changes over time. Studies based on other longitudinal data provide circumstantial evidence. Bradford et al. [53], for example, show that among US university faculty the time spent on research (excluding administrative activities within research projects), dropped by 50% from 1979 to 2005, with fundamental science being the only exception. In spite of this drop, the number of scientific papers per faculty remained constant. The authors do not measure quality trends, but it is close at hand to infer that the increasing productivity to some part has been made possible by slicing output in smaller pieces. The study did not explicitly cover MBE researchers, but there are few reasons why they would differ from other applied sciences. As reported above, respondents in our survey repeatedly pointed to performance pressures driving a diffusion of thinly sliced papers.

“In the era of pressure to publish, there has been a document circulated to and in Universities on increasing your number of citations. This includes 'salami slicing' data to increase the number of publications from a study!” (Management journal).

The preceding sections report an array of editorial activities to combat the two problems. Almost half of the respondents had started or intended to use software to scrutinize the originality of submissions. And almost 100 respondents suggested ideas to counter the marginality problem by positive measures. Taken together, the editorial experiences and activities related to misconduct and marginality indicate that the studied journals are at the center of a competition between two logics: the logic of publication performance, productivity and volume versus the Mertonian academic ethos of discovery, disinterestedness and organized skepticism [17]. Viewed from this perspective, the rise in retractions may indicate that ´organized skepticism´ is gaining ground among journal editors, although concerns remain regarding the level of undetected and unpunished misconduct. One reason for this knowledge gap is the lack of replications and cumulative theory-building in MBE-journals. Thus an examination of cites to articles published in two leading management journals found that less than 10% of the citing papers involved any tests of the ideas they cited [54]. If an MBE-paper survives the review process, the chances are very low that it will be exposed later on. Even the 10% minority in our survey reporting they had published or were in the process of publishing replications is probably overstating their performance.

Several incidents suggest that more replications should be published in the MBE-field. A high-profile case in economics is the paper on the alleged effect of high public debt on growth [55]. Efforts by master students to replicate the findings uncovered serious data flaws in the paper which undermined both its conclusions and the theoretical assumption. The critical study was published, but not in the journal which flaunted the original paper [56]. In the management field, Lepore [57] has reexamined the core cases underpinning Christensen´s theory on disruptive innovation [58]. By extending the time period of study Lepore could reveal a very different pattern than the one suggested by Christensen, which calls for a re-assessment of his theoretical framework [59]. The fact that Lepore published in a non-academic magazine raises concerns regarding the propensity of management journals to accept replications of published theories.

The salami problem can neither be exposed by retractions, nor discovered by publishing more replication studies. In our survey, editors suggested a number of ways to make the review processes more constructive, curating, connecting and commenting which would also help to crowd out marginal contributions. Several respondents indicated that editorial activities are insufficient to keep the two problems at bay and emphasized the importance of reforms in other academic institutions, in particular the systems of academic performance management. Such reforms, however, seem to be far from realization. The ambitious Dutch commission on the serial fraudster Diederik Stapel, for example, proposed a number of institutional reforms, from Ph.D. training to faculty policies, but no changes in this strategic area [44].

The comparisons of journals in different fields and indexing status detected few differences in editorial practices (see S5 to S15 Tables in the Supporting Information for more information). Observing more similarities than differences between journal fields might be explained with the historical relations and closeness of the fields. Moreover, business & management and economics program and divisions tend to be organized under the same faculties of business administration and economics at many universities. This might contribute to a process of isomorphism, where environmental and internal conditions drive journals to emulate each other [60]. The similarities across indexing classes might be surprising but the results are in line with studies of misconduct policies in the biomedical area [26, 61], which failed to find any significant differences between high- and low-impact journals. Several factors may explain the absence of such differences in our study. Irrespective of ISI status, or discipline, journals are part of the same publishing houses and exposed to the same publisher policies. Moreover, non-indexed journals tend to aspire to become ISI-indexed and adopt practices from ISI-journals. Journal editors also observe each other and try to use similar techniques in their editorial practices and practices.

## Conclusions

This paper complements previous studies on retractions in the natural sciences by focusing on management, business and economics, and by providing data on evolving practices and ideas at these journals regarding misconduct and marginality in received submissions.

Based on a search of seven major databases, the paper reports a tenfold increase of retractions in the 2005–2015 period. A survey to the 937 journals registered in these databases with identifiable contact addresses shows that the use of software tools to detect plagiarism before publication is diffusing rapidly, but also that these tools increase the editorial workload, since their output needs to be assessed intelligently. The jury is still out if the upward trend in retractions is an indicator of increasing dishonesty or increasing editorial vigilance, but the use of screening tools signifies that journals and publishers are taking the misconduct problem seriously. As for the marginality issue, more than half of the responding editors acknowledged the problem of thinly sliced salami-style papers. Some journals used software to detect this problem too, or referred to the power of the reviewing system. Others found it hard to deal with, since salami cases tend to be identified post-publication, and cannot be retracted, and publically reported.

The literature on academic identity construction illustrates how modern academics are pressured by a powerful productivity logic focused on numbers and volume [62, 63, 64]. This pressure can explain some of the problems observed, but the literature tends to overlook important countervailing forces. The activities of academic journals to detect and retract fabricated or plagiarized submissions, and their editors´ ideas to support creative contributions instead of accepting increasingly marginal papers, indicate that the productivity logic is competing head on with an invigorated Mertonian ethos of discovery and organized skepticism. Investigations of serial fraud emphasize the role of faculty policies and procedures to support whistleblowers, but so far there is little discussion of the performance metrics applied by universities. A sustainable balance between the push for high research productivity and the classical qualities embodied by academic journals [65] will probably need support from publishers as well as other institutions, including academic associations and funding agencies.

## Supporting Information

S1 Datazip Data Files. (7Z)

S1 Table Questions in the Survey II. (PDF)

S2 Table Overview of answers to open-ended questions in Survey II. (PDF)

S3 Table Diffusion of practices related to misconduct management (absolute numbers and %). (PDF)

S4 Table Journal practices related to reviewers and the review process (absolute numbers and %). (PDF)

S5 Table Cross tabulations of journal features and using software to check submissions for originality. (PDF)

S6 Table Cross tabulations of journal features and providing data files and calculations. (PDF)

S7 Table Cross tabulations of journal features and requesting corresponding authors to provide information on the specific role of each author. (PDF)

S8 Table Cross tabulations of journal features and policy regarding maximum number of papers/year authored or co-authored by any member of the editorial or advisory board. (PDF)

S9 Table Cross tabulations of journal features and experiencing any tendency of salami publishing. (PDF)

S10 Table Cross tabulations of journal features and public rewards for good reviewers. (PDF)

S11 Table Cross tabulations of journal features and using review quality instrument to engage authors in evaluating the reviewers. (PDF)

S12 Table Cross tabulations of journal features and policy to add good reviewers to the advisory board. (PDF)

S13 Table Cross tabulations of journal features and implementing any crowd-sourcing techniques to engage more reviewers. (PDF)

S14 Table Cross tabulations of journal features and publication of replication study. (PDF)

S15 Table Cross tabulations of journal features and organizing debates on a specific theme. (PDF)
