# Supplementary material for: Misconduct, Marginality and Editorial Practices in Management, Business and Economics Journals
Source: PLoS One. 2016 Jul 25;11(7):e0159492. doi: 10.1371/journal.pone.0159492 (PMC4959770; doi:10.1371/journal.pone.0159492)
Supplement: S1 Table — (PDF) [file pone.0159492.s002.pdf]

S1 Table. Questions in the Survey II

Please provide general information about your journal!

- Is your journal indexed in SSCI of Thomson Reuther (Web of Science) ISI?
  [ ] Yes [ ] No
- What is your journal area (Journal main area)?
  [ ] Business.
  [ ] Economics.
  [ ] Cross-Disciplinary (All Business & Economics Fields).
  [ ] Others please write:

Please answer the following questions with yes. no or I do not know alternatives. The following Questions related to general editorial practices!

- 1.1. Has your journal published any replication studies in the last two years?
- 1.2. Has your journal recently organized debates on a specific theme or finding?
- 1.3. Do you have any public rewards for good reviewers?
- 1.4. Do you have any policy to add good reviewers to the advisory board after specific years?
- 1.5. Do you use any review quality instrument to engage authors in evaluating the reviewers?
- 1.6 Have you tried to implement any crowd-sourcing techniques to engage more reviewers? (For example: Publicly posted submitted titles & abstracts; organized open bidding for reviewing; followed by the editor's final choice to secure the review.)
- *If you have any comment on Questions 1.1-1.6 please state here:*

Please answer the following questions with yes. no or I do not know alternatives. The following Questions related to misconduct management.

- 2.1. Do you regularly use software to check submissions for originality?
- 2.2. Does your journal have any policy regarding maximum number of papers/year authored or co-authored by any member of the editorial or advisory boards?
- 2.3. Do you ask authors to provide data files and calculations?
- 2.4. Do you experience any tendency of "salami publishing" (the slicing of output into least publishable units) in submitted papers?
- 2.5. Do you request corresponding authors to provide information on the specific role of each author? (If your answer is yes please explain us below in comment box)
- *If you have any comment on Questions* 2-1-2.5 *please state here:*

Please provide your view about how to reduce the risk and increase creativity in academic publication

2.6. Please share ideas or practices which may help to reduce the risk of dishonest papers being published:

2.7. Please share ideas or practices which may help to encourage creative and thoughtful contributions:
