# Supplementary material for: Misconduct, Marginality and Editorial Practices in Management, Business and Economics Journals
Source: PLoS One. 2016 Jul 25;11(7):e0159492. doi: 10.1371/journal.pone.0159492 (PMC4959770; doi:10.1371/journal.pone.0159492)
Supplement: S2 Table — (PDF) [file pone.0159492.s003.pdf]

S2 Table. Overview of answers to open-ended questions in Survey II

**Survey questions**	**Total number of answers**	**Recurring themes**	**Rich answers***
Comments for (1.1. – 1.6) (replications, debates. reviewer policies)	48	Reviewers (22)<br>Replications (3)<br>Crowd sourcing (3)	17
Comments for (2.1 – 2.5) (screening. data requirement; papers by editors, co-authorship, salami.	75	SW for screening (15)<br>Salami issues (13)	21
2.6 (ideas to reduce risks of dishonest publications)	163	Good reviewers (30)<br>Software (22)	57
2.7 (ideas to encourage creative papers)	98	Special issues (7)<br>Conferences/w-shops (5)<br>Change performance metrics (4)<br>See also Table 7 in the main article.	40

*Rich answer: Comments exceeding three lines in our printout qualify as ´rich´.
