# Supplementary material for: Misconduct, Marginality and Editorial Practices in Management, Business and Economics Journals
Source: PLoS One. 2016 Jul 25;11(7):e0159492. doi: 10.1371/journal.pone.0159492 (PMC4959770; doi:10.1371/journal.pone.0159492)
Supplement: S3 Table — (PDF) [file pone.0159492.s004.pdf]

S3 Table. Diffusion of practices related to misconduct management (absolute numbers and %)

	No Frequency (%)	Yes (%. Frequency)	I don't know (%. Frequency)
Do you regularly use software to check submissions for originality?	161 (54.0)	126 (42.3)	11 (3.7)
Do you ask authors to provide data files and calculations?	201 (67.5)	91 (30.5)	6 (2.0)
Do you request corresponding authors to provide information on the specific role of each author?	274 (92.0)	17 (5.7)	7 (2.3)
Does your journal have any policy regarding maximum number of papers/year authored or co-authored by any member of the editorial or advisory boards?	225 (75.5)	52 (17.5)	21 (7.0)
Do you experience any tendency of "**salami** publishing" (the slicing of output into least publishable units) in submitted papers?	124 (41.6)	154 (51.7)	20 (6.7)

^{n}= 298 journals
