# Supplementary material for: Misconduct, Marginality and Editorial Practices in Management, Business and Economics Journals
Source: PLoS One. 2016 Jul 25;11(7):e0159492. doi: 10.1371/journal.pone.0159492 (PMC4959770; doi:10.1371/journal.pone.0159492)
Supplement: S4 Table — (PDF) [file pone.0159492.s005.pdf]

S4 Table. Journal practices related to reviewers and the review process (absolute numbers and %)

	No Frequency (%)	Yes (%. Frequency)	I don't know (%. Frequency)
Do you have any **public rewards** for good reviewers?	181 (60.7)	113 (37.9)	4 (1.3)
Do you have any policy to **add good reviewers** to the advisory board after specific years?	133 (44.6)	147 (49.3)	18 (6.0)
Have you tried to implement **any crowd-sourcing** techniques to engage more reviewers?	274 (91.9)	17 (5.7)	7 (2.3)
Do you use any **review quality instrument** to engage authors in evaluating the reviewers?	232 (77.9)	59 (19.8)	7 (2.3)
Has your journal recently organized **debates** on a specific theme or finding?	148 (49.7)	143(48.0)	7 (2.3)
Has your journal published any **replication** studies in the last two years?	216 (72.5)	31 (10.4)	51 (17.1)

^{n}= 298 journals
