# Supplementary material for: Misconduct, Marginality and Editorial Practices in Management, Business and Economics Journals
Source: PLoS One. 2016 Jul 25;11(7):e0159492. doi: 10.1371/journal.pone.0159492 (PMC4959770; doi:10.1371/journal.pone.0159492)
Supplement: S5 Table — (PDF) [file pone.0159492.s006.pdf]

S5 Table. Cross tabulations of journal features and using software to check submissions for originality

A. Cross tabulation of journal main field and using software to check submissions for originality

Using software to check submissions for originality		Journal main field			Total
		**Business & Management**	**Economics**	**Cross-Disciplinary**	
	No	78	56	27	161
	% within Journal main field	51.7%	65.1%	54.0%	56.1%
	% of Total	27.2%	19.5%	9.4%	56.1%
	Yes	73	30	23	126
	% within Journal main field	48.3%	34.9%	46.0%	43.9%
	% of Total	25.4%	10.5%	8.0%	43.9%

N=287; df=2; Pearson χ^2=4.14; Likelihood Ratio χ^2=4.19; Cramer's V=0.12;
***p<.001; **p<.01; *p<.05

B. Cross tabulation of journal indexing status and using software to check submissions for originality

Using software to check submissions for originality		Journal indexing status		Total
		**Non-ISI**	**ISI**	
	No	72	89	161
	% within Journal indexing status	54.5%	57.4%	56.1%
	% of Total	25.1%	31.0%	56.1%
	Yes	60	66	126
	% within Journal indexing status	45.5%	42.6%	43.9%
	% of Total	20.9%	23.0%	43.9%

N=287; df=1; Pearson χ^2=0.24; Likelihood Ratio χ^2=0.24; Φ=-0.03

***p<.001; **p<.01; *p<.05 [Fisher's Exact Test=0.64]
