# Supplementary material for: Misconduct, Marginality and Editorial Practices in Management, Business and Economics Journals
Source: PLoS One. 2016 Jul 25;11(7):e0159492. doi: 10.1371/journal.pone.0159492 (PMC4959770; doi:10.1371/journal.pone.0159492)
Supplement: S6 Table — (PDF) [file pone.0159492.s007.pdf]

S6 Table. Cross tabulations of journal features and providing data files and calculations

A. Cross tabulation of journal main field and providing data files and calculations

**Asking authors to provide data files and calculations**		**Journal main field**			
		**Business & Management**	**Economics**	**Cross-Disciplinary**	**Total**
	No	127	38	36	201
	% within Journal main field	80.4%	45.2%	72.0%	68.8%
	% of Total	43.5%	13.0%	12.3%	68.8%
	Yes	31	46	14	91
	% within Journal main field	19.6%	54.8%	28.0%	31.2%
	% of Total	10.6%	15.8%	4.8%	31.2%

N=292; df=2; Pearson χ^2=31.85***; Likelihood Ratio χ^2=30.89***; Cramer's V=0.33***;
***p<.001; **p<.01; *p<.05

B. Cross tabulation of journal indexing status and providing data files and calculations

**Asking authors to provide data files and calculations**		**Journal indexing status**		
		**Non-ISI**	**ISI**	**Total**
	No	95	106	201
	% within Journal indexing status	71.4%	66.7%	68.8%
	% of Total	32.5%	36.3%	68.8%
	Yes	38	53	91
	% within Journal indexing status	28.6%	33.3%	31.2%
	% of Total	13.0%	18.2%	31.2%

N=292; df=1; Pearson χ^2=0.77; Likelihood Ratio χ^2=0.77; Φ=0.05
***p<.001; **p<.01; *p<.05 [Fisher's Exact Test=0.45]
