# Supplementary material for: Misconduct, Marginality and Editorial Practices in Management, Business and Economics Journals
Source: PLoS One. 2016 Jul 25;11(7):e0159492. doi: 10.1371/journal.pone.0159492 (PMC4959770; doi:10.1371/journal.pone.0159492)
Supplement: S7 Table — (PDF) [file pone.0159492.s008.pdf]

S7 Table. Cross tabulations of journal features and requesting corresponding authors to provide information on the specific role of each author

A. Cross tabulation of journal main field and requesting corresponding authors to provide information on the specific role of each author

**Requesting corresponding authors to provide information on the specific role of each author**		**Journal main field**			
		**Business & Management**	**Economics**	**Cross-Disciplinary**	**Total**
	No	147	83	44	274
	% within Journal main field	95.5%	94.3%	89.8%	94.2%
	% of Total	50.5%	28.5%	15.1%	94.2%
	Yes	7	5	5	17
	% within Journal main field	4.5%	5.7%	10.2%	5.8%
	% of Total	2.4%	1.7%	1.7%	5.8%

N=291; df=2; Pearson χ^2=2.17; Likelihood Ratio χ^2=1.91; Cramer's V=0.09
***p<.001; **p<.01; *p<.05

B. Cross tabulation of journal main field and requesting corresponding authors to provide information on the specific role of each author

**Requesting corresponding authors to provide information on the specific role of each author**		**Journal indexing status**		
		**Non-ISI**	**ISI**	**Total**
	No	123	151	274
	% within Journal indexing status	93.2%	95.0%	94.2%
	% of Total	42.3%	51.9%	94.2%
	Yes	9	8	17
	% within Journal indexing status	6.8%	5.0%	5.8%
	% of Total	3.1%	2.7%	5.8%

N=291; df=1; Pearson χ^2=0.42; Likelihood Ratio χ^2=0.42; Φ=-0.04
***p<.001; **p<.01; *p<.05 [Fisher's Exact Test=0.62]
