# Supplementary material for: Misconduct, Marginality and Editorial Practices in Management, Business and Economics Journals
Source: PLoS One. 2016 Jul 25;11(7):e0159492. doi: 10.1371/journal.pone.0159492 (PMC4959770; doi:10.1371/journal.pone.0159492)
Supplement: S8 Table — (PDF) [file pone.0159492.s009.pdf]

S8 Table. Cross tabulations of journal features and policy regarding maximum number of papers/year authored or co-authored by any member of the editorial or advisory board

A. Cross tabulation of journal main field and policy regarding maximum number of papers/year authored or co-authored by any member of the editorial or advisory board

**Policy regarding maximum number of papers/year authored or co-authored by any member of the editorial or advisory board**		**Journal main field**			
		**Business & Management**	**Economics**	**Cross-Disciplinary**	**Total**
	No	123	61	41	225
	% within Journal main field	82.6%	76.3%	85.4%	81.2%
	% of Total	44.4%	22.0%	14.8%	81.2%
	Yes	26	19	7	52
	% within Journal main field	17.4%	23.8%	14.6%	18.8%
	% of Total	9.4%	6.9%	2.5%	18.8%

N=277; df=2; Pearson χ^2=2.03; Likelihood Ratio χ^2=1.99; Cramer's V=0.08;

***p<.001; **p<.01; *p<.05

B. Cross tabulation of journal main field and policy regarding maximum number of papers/year authored or co-authored by any member of the editorial or advisory board

**Policy regarding maximum number of papers/year authored or co-authored by any member of the editorial or advisory board**		**Journal indexing status**		
		**Non-ISI**	**ISI**	**Total**
	No	100	125	225
	% within Journal indexing status	80.0%	82.2%	81.2%
	% of Total	36.1%	45.1%	81.2%
	Yes	25	27	52
	% within Journal indexing status	20.0%	17.8%	18.8%
	% of Total	9.0%	9.7%	18.8%

N=277; df=1; Pearson χ^2=0.23; Likelihood Ratio χ^2=0.23; Φ=-0.03
***p<.001; **p<.01; *p<.05 [Fisher's Exact Test=0.65]
