# Supplementary material for: Misconduct, Marginality and Editorial Practices in Management, Business and Economics Journals
Source: PLoS One. 2016 Jul 25;11(7):e0159492. doi: 10.1371/journal.pone.0159492 (PMC4959770; doi:10.1371/journal.pone.0159492)
Supplement: S9 Table — (PDF) [file pone.0159492.s010.pdf]

S9 Table. Cross tabulations of journal features and experiencing any tendency of salami publishing

A. Cross tabulation of journal main field and experiencing any tendency of "salami publishing"

**Experiencing any tendency of "salami publishing"**		**Journal main field**			
		**Business & Management**	**Economics**	**Cross-Disciplinary**	**Total**
	No	59	42	23	124
	% within Journal main field	39.9%	49.4%	51.1%	44.6%
	% of Total	21.2%	15.1%	8.3%	44.6%
	Yes	89	43	22	154
	% within Journal main field	60.1%	50.6%	48.9%	55.4%
	% of Total	32.0%	15.5%	7.9%	55.4%

N=278; df=2; Pearson χ^2=2.91 Likelihood Ratio χ^2=2.91; Cramer's V=0.10;
***p<.001; **p<.01; *p<.05

B. Cross tabulation of journal indexing status and experiencing any tendency of "salami publishing"

**Experiencing any tendency of "salami publishing"**		**Journal indexing status**		
		**Non-ISI**	**ISI**	**Total**
	No	58	66	124
	% within Journal indexing status	47.2%	42.6%	44.6%
	% of Total	20.9%	23.7%	44.6%
	Yes	65	89	154
	% within Journal indexing status	52.8%	57.4%	55.4%
	% of Total	23.4%	32.0%	55.4%

N=278; df=1; Pearson χ^2=0.58; Likelihood Ratio χ^2=0.58; Φ=0.05
***p<.001; **p<.01; *p<.05 [Fisher's Exact Test=0.49]
