# Supplementary material for: Misconduct, Marginality and Editorial Practices in Management, Business and Economics Journals
Source: PLoS One. 2016 Jul 25;11(7):e0159492. doi: 10.1371/journal.pone.0159492 (PMC4959770; doi:10.1371/journal.pone.0159492)
Supplement: S10 Table — (PDF) [file pone.0159492.s011.pdf]

S10 Table. Cross tabulations of journal features and public rewards for good reviewers

A. Cross tabulation of journal main field and public rewards for good reviewers

**Public rewards for good reviewers**		**Journal main field**			
		**Business & Management**	**Economics**	**Cross-Disciplinary**	**Total**
	No	78	69	34	181
	% within Journal main field	49.4%	81.2%	66.7%	61.6%
	% of Total	26.5%	23.5%	11.6%	61.6%
	Yes	80	16	17	113
	% within Journal main field	50.6%	18.8%	33.3%	38.4%
	% of Total	27.2%	5.4%	5.8%	38.4%

N=294; df=2; Pearson χ^2=24.31***; Likelihood Ratio χ^2=25.54***; Cramer's V=0.29***;
***p<.001; **p<.01; *p<.05

B. Cross tabulation of journal indexing status and public rewards for good reviewers

**Public rewards for good reviewers**		**Journal indexing status**		
		**Non-ISI**	**ISI**	**Total**
	No	83	98	181
	% within Journal indexing status	62.4%	60.9%	61.6%
	% of Total	28.2%	33.3%	61.6%
	Yes	50	63	113
	% within Journal indexing status	37.6%	39.1%	38.4%
	% of Total	17.0%	21.4%	38.4%

N=294; df=1; Pearson χ^2=0.07; Likelihood Ratio χ^2=0.07; Φ=0.02
***p<.001; **p<.01; *p<.05; [Fisher's Exact Test=0.81]
