# Supplementary material for: Misconduct, Marginality and Editorial Practices in Management, Business and Economics Journals
Source: PLoS One. 2016 Jul 25;11(7):e0159492. doi: 10.1371/journal.pone.0159492 (PMC4959770; doi:10.1371/journal.pone.0159492)
Supplement: S11 Table — (PDF) [file pone.0159492.s012.pdf]

S11 Table. Cross tabulations of journal features and using review quality instrument to engage authors in evaluating the reviewers

A. Cross tabulation of journal main field and using review quality instrument to engage authors in evaluating the reviewers

**Using review quality instrument to engage authors in evaluating the reviewers**		**Journal main field**			
		**Business & Management**	**Economics**	**Cross-Disciplinary**	**Total**
	No	119	72	41	232
	% within Journal main field	76.3%	84.7%	82.0%	79.7%
	% of Total	40.9%	24.7%	14.1%	79.7%
	Yes	37	13	9	59
	% within Journal main field	23.7%	15.3%	18.0%	20.3%
	% of Total	12.7%	4.5%	3.1%	20.3%

N=291; df=2; Pearson χ^2=2.61; Likelihood Ratio χ^2=2.66; Cramer's V=0.09;
***p<.001; **p<.01; *p<.05

B. Cross tabulation of journal indexing status and using review quality instrument to engage authors in evaluating the reviewers

**Using review quality instrument to engage authors in evaluating the reviewers**		**Journal indexing status**		
		**Non-ISI**	**ISI**	**Total**
	No	107	125	232
	% within Journal indexing status	81.7%	78.1%	79.7%
	% of Total	36.8%	43.0%	79.7%
	Yes	24	35	59
	% within Journal indexing status	18.3%	21.9%	20.3%
	% of Total	8.2%	12.0%	20.3%

N=291; df=1; Pearson χ^2=0.56; Likelihood Ratio χ^2=0.57; Φ=0.04
***p<.001; **p<.01; *p<.05; [Fisher's Exact Test=0.47]
