# Supplementary material for: Misconduct, Marginality and Editorial Practices in Management, Business and Economics Journals
Source: PLoS One. 2016 Jul 25;11(7):e0159492. doi: 10.1371/journal.pone.0159492 (PMC4959770; doi:10.1371/journal.pone.0159492)
Supplement: S12 Table — (PDF) [file pone.0159492.s013.pdf]

S12 Table. Cross tabulations of journal features and policy to add good reviewers to the advisory board

A. Cross tabulation of journal main field and policy to add good reviewers to the advisory board

**Policy to add good reviewers to the advisory board after specific years**		**Journal main field**			
		**Business & Management**	**Economics**	**Cross-Disciplinary**	**Total**
	No	53	53	27	133
	% within Journal main field	35.1%	65.4%	56.3%	47.5%
	% of Total	18.9%	18.9%	9.6%	47.5%
	Yes	98	28	21	147
	% within Journal main field	64.9%	34.6%	43.8%	52.5%
	% of Total	35.0%	10.0%	7.5%	52.5%

N=280; df=2; Pearson χ^2=21.23***; Likelihood Ratio χ^2=21.51***; Cramer's V=0.28***; ***p<.001; **p<.01; *p<.05

B. Cross tabulation of journal indexing status and policy to add good reviewers to the advisory board

**Policy to add good reviewers to the advisory board after specific years**		**Journal indexing status**		
		**Non-ISI**	**ISI**	**Total**
	No	56	77	133
	% within Journal indexing status	44.4%	50.0%	47.5%
	% of Total	20.0%	27.5%	47.5%
	Yes	70	77	147
	% within Journal indexing status	55.6%	50.0%	52.5%
	% of Total	25.0%	27.5%	52.5%

N=280; df=1; Pearson χ^2=0.86; Likelihood Ratio χ^2=0.86; Φ=-0.05

***p<.001; **p<.01; *p<.05; [Fisher's Exact Test=0.40]
