# Supplementary material for: Misconduct, Marginality and Editorial Practices in Management, Business and Economics Journals
Source: PLoS One. 2016 Jul 25;11(7):e0159492. doi: 10.1371/journal.pone.0159492 (PMC4959770; doi:10.1371/journal.pone.0159492)
Supplement: S13 Table — (PDF) [file pone.0159492.s014.pdf]

S13 Table. Cross tabulations of journal features and implementing any crowd-sourcing techniques to engage more reviewers

A. Cross tabulation of journal main field and implementing any crowd-sourcing techniques to engage more reviewers

**Implementing any crowd-sourcing techniques to engage more reviewers**		**Journal main field**			
		**Business & Management**	**Economics**	**Cross-Disciplinary**	**Total**
	No	142	82	50	274
	% within Journal main field	91.6%	96.5%	98.0%	94.2%
	% of Total	48.8%	28.2%	17.2%	94.2%
	Yes	13	3	1	17
	% within Journal main field	8.4%	3.5%	2.0%	5.8%
	% of Total	4.5%	1.0%	0.3%	5.8%

N=291; df=2; Pearson χ^2=4.05; Likelihood Ratio χ^2=4.43; Cramer's V=12;
***p<.001; **p<.01; *p<.05

B. Cross tabulation of journal indexing status and implementing any crowd-sourcing techniques to engage more reviewers

**Implementing any crowd-sourcing techniques to engage more reviewers**		**Journal indexing status**		
		**Non-ISI**	**ISI**	**Total**
	No	128	146	274
	% within Journal indexing status	95.5%	93.0%	94.2%
	% of Total	44.0%	50.2%	94.2%
	Yes	6	11	17
	% within Journal indexing status	4.5%	7.0%	5.8%
	% of Total	2.1%	3.8%	5.8%

N=291; df=1; Pearson χ^2=0.84; Likelihood Ratio χ^2=0.86; Φ=0.05
***p<.001; **p<.01; *p<.05; [Fisher's Exact Test=0.46]
