# Supplementary material for: Misconduct, Marginality and Editorial Practices in Management, Business and Economics Journals
Source: PLoS One. 2016 Jul 25;11(7):e0159492. doi: 10.1371/journal.pone.0159492 (PMC4959770; doi:10.1371/journal.pone.0159492)
Supplement: S14 Table — (PDF) [file pone.0159492.s015.pdf]

S14 Table. Cross tabulations of journal features and publication of replication study

A. Cross tabulation of journal main field and publication of replication study

**Publishing replication study**		**Journal main field**			
		**Business & Management**	**Economics**	**Cross-Disciplinary**	**Total**
	No	114	62	40	216
	% within Journal main field	85.7%	89.9%	88.9%	87.4%
	% of Total	46.2%	25.1%	16.2%	87.4%
	Yes	19	7	5	31
	% within Journal main field	14.3%	10.1%	11.1%	12.6%
	% of Total	7.7%	2.8%	2.0%	12.6%

N=247; df=2; Pearson χ^2=0.81; Likelihood Ratio χ^2=0.83; Cramer's V=0.06
***p<.001; **p<.01; *p<.05

B. Cross tabulation of journal indexing status and publication of replication study

**Publishing replication study**		**Journal indexing status**		
		**Non-ISI**	**ISI**	**Total**
	No	107	109	216
	% within Journal indexing status	90.7%	84.5%	87.4%
	% of Total	43.3%	44.1%	87.4%
	Yes	11	20	31
	% within Journal indexing status	9.3%	15.5%	12.6%
	% of Total	4.5%	8.1%	12.6%

N=247; df=1; Pearson χ^2=2.14; Likelihood Ratio χ^2=2.18; Φ=0.09
***p<.001; **p<.01; *p<.05; [Fisher's Exact Test=0.18]
