# Supplementary material for: Misconduct, Marginality and Editorial Practices in Management, Business and Economics Journals
Source: PLoS One. 2016 Jul 25;11(7):e0159492. doi: 10.1371/journal.pone.0159492 (PMC4959770; doi:10.1371/journal.pone.0159492)
Supplement: S15 Table — (PDF) [file pone.0159492.s016.pdf]

S15 Table. Cross tabulations of journal features and organizing debates on a specific theme

A. Cross tabulation of journal main field and organizing debates on a specific theme

Organizing debates on a specific theme		Journal main field			
		Business & Management	Economics	Cross-Disciplinary	Total
	No	81	37	30	148
	% within Journal main field	52.9%	42.5%	58.8%	50.9%
	% of Total	27.8%	12.7%	10.3%	50.9%
	Yes	72	50	21	143
	% within Journal main field	47.1%	57.5%	41.2%	49.1%
	% of Total	24.7%	17.2%	7.2%	49.1%

N=291; df=2; Pearson χ^2=3.98; Likelihood Ratio χ^2=3.99; Cramer's V=0.12;
***p<.001; **p<.01; *p<.05

B. Cross tabulation of Journal Indexing status and organizing debates on a specific theme

Organizing debates on a specific theme		Journal indexing status		
		Non-ISI	ISI	Total
	No	67	81	148
	% within Journal indexing status	50.4%	51.3%	50.9%
	% of Total	23.0%	27.8%	50.9%
	Yes	66	77	143
	% within Journal indexing status	49.6%	48.7%	49.1%
	% of Total	22.7%	26.5%	49.1%

N=291; df=1; Pearson χ^2=0.02; Likelihood Ratio χ^2=0.02; Φ=-0.01
***p<.001; **p<.01; *p<.05; [Fisher's Exact Test=0.91]
